# Rethinking Eldercare in a Digital Age: Internet Use and Shifting Attitudes in China

**DOI:** 10.1093/geroni/igaf122.4358

**Published:** 2025-12-31

**Authors:** Mengting Li, Zhiyong Lin, Yibo Li

**Affiliations:** Renmin University of China, Beijing, Beijing, China (People’s Republic); University of Texas at San Antonio, San Antonio, Texas, United States; Renmin University of China, Beijing, Beijing, China (People’s Republic)

## Abstract

This study examines how internet use is associated with older adults’ attitudes and preferences regarding eldercare in China. Specifically, it explores whether internet access and usage patterns are linked to views on who should be primarily responsible for caring for older adults and to preferences for informal, formal, or mixed care arrangements. Data come from the 2020 wave of the China Longitudinal Aging Social Survey (CLASS), a nationally representative survey of community-dwelling adults aged 60 and above. Multinomial logistic regression models were used to assess the associations between four indicators of internet use (access, frequency, number of online activities, and digital proficiency) and two outcome measures: (1) perceived responsibility for eldercare (family, government/society, or shared), and (2) personal care preferences (family-based caregiving, community-based services or institutional care). Internet access was associated with lower support for formal care but greater endorsement of shared responsibility, compared to family-only care. Greater online activities and digital proficiency were positively associated with support for shared responsibility. In terms of care preferences, internet access was negatively associated with preference for community-based services and institutional care, whereas online activity and proficiency were positively associated with such preferences. Digitally engaged individuals were more likely to consider formal care than non-users, though family care remained the most preferred option. Internet use is linked to more nuanced and flexible views on eldercare among older Chinese adults. Digital engagement may play a role in reshaping traditional expectations and expanding consideration of formal care in aging societies.

